# Cook County Medical Society

**Published:** 1853-08

**Authors:** Marshall Hall


*Cook Co. Medical Society, July 5th.*—Notes of Experiments performed by Marshall Hall, M.D., F. R. S.; reported by J. W. Freer, M.D., Demonstrator of Anatomy in Rush Medical College.

No. 1.—The spinal cord of a frog was severed as nearly as possible at the upper extremity of the medulla oblongata, after which, for a few minutes, no movements could be produced by irritation—probably owing to the shock of the operation—but upon recovery from the depression which lasted from two to three minutes, upon drawing out the posterior extremities, the animal withdrew them forcibly; again upon drawing out one posterior extremity and gently removing the hand, so as to cause the least possible irritation, there was no spontaneous disposition to contraction; but upon the application of excitement, by rubbing or pinching the toes, the extremity contracted with force. Even the slight irritation produced by the jarring of the table, or the drying of the water from the surface of the animal, was sufficient to produce motion. Again the animal was suspended by one foot, also placed across the edge of a tumbler, and in other painful positions, without exhibiting a disposition to spontaneous movement, at least while those present avoided jarring the furniture upon which it was placed.

No. 2.—The spinal cord was divided between the cerebrum and medulla oblongata, the same phenomena as were observed in No. 1, were produced, after which the viscera and head were removed. The head with the cerebrum, the viscera with the ganglionic system and trunk with the spinal system of nerves being placed in separate positions, the trunk still moved and leaped upon the table upon the application of excitement; but it was observed that motion mas excited more readily by the natural touch, than by pricking or cutting.

The next experiment was the removal of the skin from one of the posterior extremities, and the result was, that no kind of irritation upon the denuded surface was capable of producing contractions, while the same stimulus upon the opposite extremity produced active movements, hence it was concluded that the origin of the nerves of incidence must be in the skin. Next the lumbar nerve of the opposite side from the denuded extremity was severed, after which, no motion could be excited upon that side, but motion could still be produced upon the extremity from which the skin had been removed by irritating the surface above the denuded portion, also by pinching the lumbar plexus of the same side. The spinal marrow was then broken down, after which no movements could be produced.

*Query.*—After the division of the spinal cord at its junction with the encephalon, in the above experiments, was there still sensation and volition remaining? The phenomena observed seem to give a decided negative.

In animal life every act or movement is generally accompanied by two things, viz: brain action and spinal action; ordinarily they are simultaneous in their manifestations, for instance, the eye may be closed by volition, it is also closed involuntarily by the approach or contact of foreign bodies; in this instance it is closed by spinal action alone; the two failing to agree, the function is performed without the interposition of the will.

What are the facts which go to prove, that after the removal or separation of the brain from the spinal system, there is no sensation or volition remaining? The first is this: in the human subject paralyzed by injury or destruction of the spinal cord we have testimony, that there is neither sensation nor volition, and yet motion in the paralysed portion may be produced by external means.

Again, if we take an animal—the frog or snake, for example—remove the head, and place the animal in any painful position, there would be motion providing volition and sensation were remaining, yet no such movement occurs, as the above examples attest; still on fresh excitation contractions will follow.

Since the above experiments, the following have been made upon snakes.

No. 1.—The spinal column was divided at a point near the anus, the animal's movements were but slightly interfered with, for the excitement produced by the movement of the anterior portion imparted sufficient stimulus to the posterior division to cause simultaneous motion throughout the body.

Next the spinal column was severed from the encephalon, after which the same results were obtained as with the frog, viz: upon suspending it by the tail, as soon as the excitement derived from the handling in order to place it in such position had passed away, no motion occurred for the space of about ten minutes, when it was again placed upon the table, the contact with the surface of which produced vigorous movements, which soon subsided into immobility as before.

No. 2.—In this instance, the first experiment was the division of all the soft parts down to the spinal column at a point near the middle of the animal. The shock was slight, and the movements seemingly not interfered with in the least.

Secondly. The spinal column was divided at about two inches back of the head, the same results followed as in the former instance. Lastly, all sources of excitement being removed, and the animal having become motionless, the position was sketched with a pencil in order to ascertain whether any movements might occur during the night with the following result: all that portion behind the point of separation had remained stationary, the anterior portion having changed its position.

